# Notes and News

**Published:** 1899-10-07

**Authors:** 


### Notes and News.

(Collected and Communicated.)

By the London School of Tropical Medicine, which opened on October 2nd, a travelling scholarship of £300 is offered.

Lectures free to medical practitioners will be given at the Hospital for Sick Children on Thursdays at four o'clock on October 12th and 26th by Dr. Penrose, and on October 19th by Dr. F. E. Batten.

We understand that the services of several medical men recently connected with our large hospitals, who have volunteered for South Africa, have been accepted by the Government, and that they will shortly proceed to Natal, where they will be employed in the base hospitals.

The Chelsea Hospital for Women will be reopened to patients on Monday next, 9th inst. The operating theatre has been enlarged, and with its adjacent rooms brought into condition to fulfil all the requirements of modern surgery. An expenditure of £3,000 has been incurred for these requirements, of which half has been already subscribed.

The Metropolitan Hospital Saturday collection will take place on October 14th. The collection will be made in the same manner which has been adopted since the street collection has been abandoned in response to the general public feeling against it. It is to be hoped that this excellent institution will be well supported on the present occasion. We are glad to report that the receipts from business houses are considerably larger than last year.

The foundation-stone for a borough isolation hospital at Reigate has been laid by the Mayor. The site is sufficiently remote from the populous part of the town, but is conveniently reached by good roads from all directions. The administrative block will be three storeys high. There are to be separate pavilions for scarlet fever, diphtheria, and isolation cases. Cases of typhoid, as well as doubtful cases, will be housed in the last. The cost is estimated at £16,000.

Plague still continues in Oporto. It does not seem to be very severe, but it spreads, having now appeared at a village outside the sanitary cordon.

The Royal Infirmary and the Western Infirmary, Glasgow, have both received splendid legacies of upwards of £13,000 each through the will of the late Mr. Alexander Boyack.

A new degree, that of Doctor of Pharmacy, has been conferred by the University of Paris. The first recipient of the honour is Mons. La Court, who took for his thesis the subject of the water of Versailles.

The annual dinner of the old students of the London Hospital was held on October 2nd in the College Library. Dr. G. E. Herman occupied the chair, and a considerable number of old students and guests were present.

It is reported from Sierra Leone that those who are engaged investigating the mosquito theory of malaria in that district poured a drachm of kerosene oil, as an experiment, upon a puddle about a square yard in area, floating on which were a number of *Anopheles* larvæ, with the result that after six hours all the larvæ were found to be dead.

The death occurred last Saturday of Surgeon-General Sir Charles A. Gordon, K.C.B., Honorary Physician to the Queen. He was born in 1821, and became assistant-surgeon to the Buffs in 1841, and from that time forwards saw much active service. During the Franco-German War he was sent by the War Office as Medical Commissioner to the French Army, and was in Paris throughout the siege and bombardment.

At the Annual Medical Service in State of the Guild of St. Luke, which will take place in St. Paul's Cathedral on October 19th at half-past seven p.m., the sermon will be preached by the Bishop of Stepney. The Lord Mayor will attend in state, and it is expected that many University Graduates and others having the right to do so will appear in their robes. The music will be rendered by a choir of over 200 voices, provided by the London Gregorian Association. Last year upwards of 1,200 medical men attended the service. Admission is by ticket only, and all information can be obtained from the Registrar, 24, Somerset Street, Portman Square, W.

The Leicester Guardians have made up their minds. A special meeting was held on Monday last to consider the *mandamus* of the High Court of Justice peremptorily ordering them to appoint a vaccination officer. Forty-seven members attended, only one being absent. A considerable number of the Guardians favoured the appointment of an officer, and of their enforcing the claim of the board to control his action. After a long discussion it was decided by 28 votes to 18 not to appoint an officer, and to take the consequences. What the consequences will be is not as yet quite clear, for it seems that in the *mandamus* there is a statement to the effect that the health of the town had suffered in consequence of the non-appointment. Now the recalcitrants say that this is not true in fact. Hence it would seem that they hope still to have something to argue about.

